# Metabolomics and Network Pharmacology-Based Investigation into the Mechanisms Underlying the Therapeutic Effect of a New Chinese Traditional Medicine (Cui Nai Ling) on Bromocriptine-Induced Hypogalactia

**DOI:** 10.1155/2021/8857449

**Published:** 2021-06-16

**Authors:** Xianglong Meng, Chenzi Lyu, Junnan Ma, Xiaoyan Zhang, Cong Hu, Xiaojuan Su, Chenxu Ning, Wenbin Xie, Shuosheng Zhang

**Affiliations:** ^1^Experimental Teaching Center, College of Chinese Materia Medica and Food Engineering, Shanxi University of Chinese Medicine, Jinzhong 030619, China; ^2^Department of Formulaology, Institute of Integrative Medicine, Dalian Medical University, Dalian 116044, China; ^3^School of Pharmacy, Jiangxi Science and Technology Normal University, Nanchang 330013, China

## Abstract

As a traditional veterinary medicine to promote lactation, Cui Nai Ling (CNL) can not only increase milk supply and promote health but also improve the overall physiological function and immunity of the animals. In order to further improve CNL's effect on lactation, we have previously made a new CNL (NCNL) by adding Tetrapanacis Medulla and replacing Vaccariae Semen with fried Vaccariae Semen in CNL. We have demonstrated that the lactation-promoting effect of NCNL is better than that of CNL. However, the underlying mechanisms by which NCNL promotes lactation are unclear. In this study, we performed metabolomics, network pharmacology, and pharmacodynamic studies to explore the underlying mechanisms by which NCNL promotes lactation in rats with bromocriptine-induced hypogalactia. The results showed that NCNL significantly improved the loss of appetite in female adult rats and the weight loss of pups caused by the disorder of lactation. Biochemical analysis showed that NCNL could regulate the levels of PRL, T4, E2, Ca, UREA, GLU, ALT, AST, TCHO, and TG in serum. The pathological results showed that NCNL could promote lactation and increase the mammary gland index by improving breast acinar tissue morphology in rats with hypogalactia. Network pharmacology studies showed that NCNL promotes lactation through P13K-Akt, insulin resistance, and prolactin signaling pathways, among which the most frequently affected pathway was the P13K-Akt signaling pathway. Metabolomics studies showed that NCNL can significantly upregulate phenylalanine, tyrosine, and tryptophan biosynthesis and tyrosine metabolism pathways and downregulate cysteine and methionine metabolism pathways. NCNL can significantly increase the serum prolactin concentration, improve the glucose and lipid metabolism disorders, and regulate PI3K-Akt, insulin resistance, and prolactin pathways to affect the amino acids' metabolism in the mammary gland and ultimately exert its therapeutic effect on bromocriptine-induced postpartum hypogalactia. These findings revealed the effect and application value of NCNL on animals with postpartum hypogalactia.

## 1. Background

China is one of the important origins of plant cultivation, animal breeding, and medicine in the world. As early as in the Western Zhou Dynasty (BC 1046∼256), there were veterinarians who began to dedicate themselves to the medical treatment of domestic animals. The “Slips on the Flowing Sands” and “Ju-Yan Han Bamboo” in the Han Dynasty (BC 202 ∼AD 220) and the “Healing Horse Prescription” and “An Ji Ji” in the Sui Dynasties (AD 581∼618) and Tang Dynasties (AD 618∼907) were precious achievements during the early development of veterinary medicine in ancient China. After the 1970s, China's veterinary medicine and veterinary drugs have advanced rapidly and its application fields expanded significantly. The production of veterinary chemical drugs and antibiotics has increased rapidly. The veterinary chemicals were characterized by “treatment with various dosages” and “rapid and definite curative effects” at that time [1, 2]. Recently, with the implementation of national veterinary drug standards, a large number of chemical drugs and antibiotics have been restricted or banned. Therefore, more and more attention has been paid to the Chinese veterinary drugs due to their advantages of low toxicity, low residue, and reliable efficacy, which has further promoted the rapid development and application of Chinese veterinary drugs in the veterinary drug industry.

In the 2015 edition of “Chinese Veterinary Pharmacopoeia”, the ingredients of Cui Nai Ling (CNL) included Vaccariae Semen, Astragali Radix, Gleditsiae Spina, Angelicae Sinensis Radix, Codonopsis Radix, Chuanxiong Rhizoma, Rhapontici Radix, and Liquidambaris Fructus [3]. CNL is a gray powder with fragrance and sweet taste. It has the effects of invigorating Qi (gas), blood and the spleen, increasing appetite, and eliminating food accumulation. Clinically, it is mainly used to treat livestock, especially hypogalactia or agalactia in dairy cows due to weak physique or postpartum spleen deficiency, lack of food, lack of Qi and blood, obstruction of meridians, and diarrhea. It can promote breast development, increase milk production and quality, and enhance immune function. In our previous studies, we have made a new CNL (NCNL) by adding Tetrapanacis Medulla and replacing Vaccariae Semen with fried Vaccariae Semen in CNL to increase its lactation-promoting effect. The preparation of NCNL was optimized according to the drying temperature, drying time, and smashing time as investigative factors and the sieving rate of the No. 2 sieve as the response parameter. However, the underlying mechanisms by which NCNL promotes lactation are unclear. In this study, we performed metabolomics, network pharmacology, and pharmacodynamic studies to explore the underlying mechanisms by which NCNL promotes lactation in rats with bromocriptine-induced hypogalactia. The results obtained in these studies were expected to provide theoretical bases for the clinical application of NCNL.

## 2. Materials and Methods

### 2.1. Experimental Herbal Medicine

Fried Vaccariae Semen, Astragali Radix, Gleditsiae Spina, Radix Angelicae Sinensis, Radix Codonopsis, Rhizoma Chuanxiong, Radix Rhapontici, Liquidambaris Fructus, and Medulla Tetrapanacis were purchased from Shanxi Herentang Chinese Medicine Decoction Pieces Co., Ltd., and were verified as authentic products by the Shanxi University of Chinese Medicine. Traditional CNL was purchased from Shanxi Haodong Tongda Biotechnology Co., Ltd. (batch number: 20190713). ELISA Kits for analysis of PROG, E2, T3, T4, and GH were purchased from Shanghai Renjie Biotechnology Co., Ltd., and the catalog numbers were RJ16569, RJ15537, RJ16177, RJ15838, and RJ16212, respectively. ELISA Kits for analysis of PRL were purchased from AndyGene Biotechnology Co., Ltd., and the catalog number was AD20190719.

### 2.2. NCNL

Fried Vaccariae Semen (20 g), Astragali Radix (10 g), Gleditsiae Spina (10 g), Radix Angelicae Sinensis (20 g), Radix Codonopsis (10 g), Rhizoma Chuanxiong (20 g); Radix Rhapontici (5 g), Liquidambaris Fructus (5 g), and Medulla Tetrapanacis (10 g) were smashed for 6 min after 4 hours of drying at 70°C. The powder was sieved through the No. 2 sieve and stored at 4°C for subsequent uses.

### 2.3. Preparation of the Aqueous Extract of CNL and NCNL

A proper amount of CNL and NCNL powder was added with 10 times of the distilled water and soaked for 30 minutes. After heating and refluxing for 1 hour, it was filtered and added with 8 times of distilled water to reflux and extract the residue for 1 h. Following filtering, the two filtrates were combined. CNL was concentrated to 1 g/mL, and NCNL was concentrated to 0.5 g/mL and 1 g/mL, respectively (the content of ferulic acid was 0.53 mg/g, the content of Senkyunolide I was 0.25 mg/g, the content of chlorogenic acid was 0.24 mg/g, and the content of ligustilide was 15.42 mg/g).

### 2.4. Animal Grouping and Drug Administration

SD pregnant rats (at 15-16 days of gestation) were purchased from SPF (Beijing) Biotechnology Co., Ltd., and raised in a single cage at the condition of 12 hours of light per day, the temperature of 22 ± 3°C, and humidity of 60 ± 5% for 7 days. The female rats within 12 hours of parturition were randomly divided into 5 groups: the normal group, the control group, CNL treatment group (CNL, 1000 mg/kg), low-dose NCNL treatment group (NCNLL, 500 mg/kg), and high-dose NCNL treatment group (NCNLH, 1000 mg/kg). Each group had 7 female rats, and each female adult was assigned 10 pups. Except for the normal group, rats in each group were treated with bromocriptine (1.6 mg/kg) by intragastric administration once a day to create a hypogalactia model. The female rats in the CNL group, NCNLL group, and NCNLH group were administered intragastrically with CNL (1000 mg/kg), low-dose NCNL (500 mg/kg), and high-dose NCNL (1000 mg/kg) at 8 am and 8 pm continuously for 7 days. Rats in the normal and control group were given the same volume of normal saline similarly. All animals were handled according to the animal welfare guidelines and approved by the Institutional Animal Care and Use Committee of Shanxi University of Chinese Medicine (2019LL202).

### 2.5. Analysis of Physical Signs

During the first week after drug administration, the weight of each female adult and the total weight of pups in each group were measured. The food and water intake of each female adult rat was monitored daily. One week after the drug administration, the adults fasted for 12 hours and were anesthetized, and blood was collected from the abdominal aorta for later uses. The mammary gland tissues of the female adult rats were collected, the thymus tissues of each adult rat were weighed, and the mammary gland index was calculated (thymus weight/adult weight ×100%).

### 2.6. Analysis of Serum Biochemical Parameters

One week after the drug administration, the blood collected from the abdominal aorta of the adult rats was centrifuged at 3000 rpm for 30 minutes, and the serum was transferred to a new EP tube. ELISA was performed to measure the concentration of prolactin (PRL), progesterone (PROG), estradiol (E2), triiodothyronine (T3), thyroxine (T4), and growth hormone (GH), and the ranges of detection were 30–800 ng/L, 0–16 ng/mL, 0–240 pg/mL, 0–10 ng/mL, 0–240 ng/mL, and 0–24 ng/mL, respectively. A biochemical automatic analyzer was used to measure the concentration of insulin (INS), insulin-like growth factor-1 (IGF-1), dopamine (DA), alanine aminotransferase (ALT), aspartate aminotransferase (AST), blood glucose (GLU), total cholesterol (TCHO), triglyceride (TG), calcium (Ca), and urea (UREA).

### 2.7. Histopathological Observation

Mammary gland tissues were fixed in 4% paraformaldehyde in 0.1 M PBS, embedded in paraffin, and cut into 5 *μ*m sections. To observe structural damage to mammary gland tissue, sections were stained with hematoxylin and eosin (H&E). The sections were visualized using light microscopy, and digital images were captured and analyzed.

### 2.8. Western Blotting Analysis

Mammary gland tissues were isolated by using a T-PER tissue protein extraction reagent (Thermo Fisher Scientific, Waltham, MA, USA). The protein content was measured using a Bradford assay, separated using SDS-PAGE, and transferred to a nitrocellulose membrane [4]. The membranes were incubated in 5% skimmed milk, dissolved in Tris-buffered saline with 1% Tween-20 (TBST), pH 7.5, for 30 min at RT for blocking, and then incubated with anti-prolactin (Boster Biological Technology Co., Ltd. Wuhan, Hubei, China), albumin (Abcam, Cambridge, England, UK), and *β*-actin (Bioswamp Biological Pharmaceutical Co., Ltd, Hubei, Wuhan, China) antibodies at 1 : 1000 overnight at 4°C and incubated with HRP-conjugated mouse or rabbit secondary antibodies for 3 h at RT. Blots were scanned and analyzed with ChemiDoc MP Imaging System (GeneGnome XRQ Gene Co., Ltd., USA).

### 2.9. Metabolomic Profiling [5]

Serum samples were thawed at 4°C on ice. Then, 100 *μ*L of the sample was taken and placed in an EP tube, extracted with 400 *μ*L of extraction solvent (methanol: acetonitrile = 1 : 1 [v/v], containing an internal standard, L-2 chlorophenylalanine at 2 *μ*g/mL), vortexed for 30 s, treated with ultrasound for 5 min (during incubation in ice water), and incubated for 1 h at −20°C to precipitate proteins. The solutions were then centrifuged at 12000 rpm for 15 min at 4°C. The supernatant was transferred into fresh EP tubes, and the extracts were dried in a vacuum concentrator without heating and reconstituted in 200 *μ*L of the extraction solvent (acetonitrile: water = 1 : 1 [v/v]). The reconstituted solution was vortexed for 30 s, sonicated for 10 min (in a 4°C water bath), and centrifuged for 15 min at 12000 rpm at 4°C. The supernatant was transferred into a fresh 2 mL LC-MS glass vial, 10 *μ*L aliquots from each sample were collected and pooled as QC samples, and the supernatant was collected for UHPLC-QTOF-MS analysis.

LC-MS/MS analyses were performed using a UHPLC system (Thermo Fisher Scientific, Waltham, MA, USA) with a Thermo C18 column (1.9 *μ*m; 2.1 × 50 mm). The mobile phase consisted of acetonitrile (A) and deionized water with 0.1% methanoic acid (B), and the following elution gradient was used: 0–1 min, 5%–5% A; 1–5 min, 5%–15% A; 5–7 min, 15%–15% A; 7–14 min, 15%–100% A; 14–19 min, 100%–100% A; 19–20 min, 100%–5% A; 20–22 min, 5%–5% A. The mobile phase was delivered at 0.3 mL min^−1^, and the injection volume was 3 *μ*L.

The Triple TOF mass spectrometer was used for its ability to acquire MS/MS spectra on a full MS dd-ms2 during the LC-MS experiment. In this mode, the electrospray capillary voltage was 3.2 kV in positive and negative ionization modes, the atomized gas velocity was 35 L·min^−1^, the auxiliary air velocity was 5 L·min^−1^, the ion source temperature was 320°C, and the auxiliary heater temperature was 350ºC. The NCE values of energy collision were 25, 35, and 45, respectively.

The LC/Q/TOF-MS metabolomic profiling data were imported into Compound Discoverer 3.0 to perform the metabolic feature extraction by the adoption of a molecular feature extraction algorithm (Thermo Fisher, Inc., Santa Clara, CA, USA). The parameters were set as follows: mass range, 100–1, 500; mass deviation, 5 × 10^−6^; retention time deviation, 0.05 min; SNR threshold, 3. SIMCA-P (Version 14.1, Umetrics AB, Umea, Sweden) was used for multivariate statistical analysis of the integral values obtained from LC-MS findings. The mean-centered data were used for principal component analysis (PCA). The modeling of sample classes was performed using orthogonal projection to latent structure discriminant analysis (OPLS-DA) algorithm at a unit variance-scaled modality. OPLS-DA analysis was performed between the two groups to evaluate the differences between the normal and the control and the control and the NCNLH and to generate corresponding *S*-plots and VIP graphs. The points at each end of the *S*-plot diagrams are potential deferentially abundant metabolites. Metabolites with VIP >3 and one-way ANOVA values with *P* < 0.05 were used to identify differences in metabolite levels between groups. The disturbed metabolites and metabolic pathways were identified by open database sources, including the Human Metabolome Database, KEGG, and MetaboAnalyst 5.0 (https://www.metaboanalyst.ca/)

### 2.10. Network Pharmacology-Based Analysis

#### 2.10.1. Screening of Active Ingredients

Chemical ingredients and the targets of BHID were obtained from the Traditional Chinese Medicine Systems Pharmacology database (TCMSP, https://tcmspw.com/tcmsp.php), a Bioinformatics Analysis Tool for Molecular mechanism of Traditional Chinese Medicine (BATMAN-TCM, http://bionet.ncpsb.org.cn/batman-tcm/), and the relevant literature. At present, there were no relevant literature studies reported on the screening of active ingredients in NCNL and their targets. This study aimed to screen all the active ingredients in NCNL to construct an “ingredient-target-disease” network and explore the pharmacodynamic mechanisms of NCNL. Therefore, the active ingredients for NCNL were not screened based on Oral Bioavailability (OB) and Drug-likeness (DL).

#### 2.10.2. Identification of NCNL and Lactation Overlapping Genes

Genes related to lactation were identified from online Mendelian Inheritance in Man (OMIM, http://www.omim.org/) [6] and GeneCards® (The Human Gene Database) (https://www.genecards.org) [7] using relevant keywords as queries. The results of the OMIM database and GeneCards database were combined according to the relevant search conditions to obtain gene targets for lactation. Drug and disease target genes were uploaded to the online Venn program (http://bioinformatics.psb.ugent.be/webtools/Venn) and obtain the overlapping genes related to the active ingredients and lactation.

#### 2.10.3. Construction of the “Ingredient-Target-Disease” Interactive Network

Gene names corresponding to the target proteins were identified according to the Universal Protein Resource (UniProt, https://www.uniprot.org) [8], the Human Genome Database (HUGO Gene Nomenclature Committee, HGNC, https://www.genenames.org/) [9], and Medical Literature Search Service System (US National Library of Medicine National Institutes of Health, PubMed, https://www.ncbi.nlm.nih.gov/pubmed) [10]. Cytoscape 3.7.1 (http://www.cytoscape.org/) software was used to construct the “ingredient-target-disease” network.

#### 2.10.4. Construction of the NCNL and Lactation Protein-Protein Interaction (PPI) Network

PPI was constructed by the protein interaction database (STRING 11.0, https://string-db.org) [11]. The confidence score threshold was set at 0.95 to hide unconnected nodes in the PPI network.

#### 2.10.5. Pathway Analysis of Core Targets

The common protein targets of NCNL and lactation were uploaded to the Database for Annotation (Visualization and Integrated Discovery, DAVID, https://david.ncifcrf.gov/home.jsp) [12]. Gene Ontology (GO) [13], biological process enrichment analysis, and Kyoto Encyclopedia of Genes and Genomes (KEGG) [14] analyses were performed.

Cytoscape 3.7.1 (http://www.cytoscape.org) [15] software was used to visualize the results.

### 2.11. Statistical Analysis

Data analyses were conducted using GraphPad Instat software (ver. 5.0; GraphPad Software, La Jolla, CA, USA). The data were expressed as the mean ± standard deviation (SD) of seven mice in each group. The significance of treatment effects was determined by one-way analysis of variance (ANOVA) followed by Tukey's post hoc analysis. The *P* value of 0.05 was considered to indicate statistical significance.

## 3. Results

### 3.1. Effect of NCNL on the Physiological Signs of Rats with Postpartum Hypogalactia

As shown in Table 1, there was no significant difference in the body weight of adult rats between the normal and control groups (*P* > 0.05). Furthermore, there was no significant difference in the body weight of adult rats between the control group and the treatment groups (CNL, NCNLL, and NCNLH) (*P* > 0.05). These results indicated that body weight of the adult rats did not change significantly after postpartum hypogalactia was induced or after CNL, NCNLL, and NCNLH administration. During the one-week drug administration period, the total weight of the pups in the control group decreased significantly from day 2 to day 7 compared with that in the normal group due to the obstruction of lactation in adult rats (*P* < 0.05). At day 7, the total weight of pubs in the CNL group was significantly higher than that in the control group (*P* < 0.05). Compared with the control group, the total weight of the pups in the NCNLL and NCNLH groups began to increase significantly at Day 5 and Day 6 (*P* < 0.05), respectively, after intragastric administration of NCNL. These results indicated that NCNLL and NCNLH can significantly improve the weight loss of pups caused by the impaired lactation of the adult rats.

In addition, during the one-week drug administration, the food intake of the adult rats in the control group was significantly lower than that in the normal group (*P* < 0.05). Food intake of the adult rats in the CNL group was not significantly different from that in normal group (*P* > 0.05). Compared to the control group, food intake in NCNLL and NCNLH groups began to increase significantly on day 5 (*P* < 0.05). These results indicated that NCNLL and NCNLH can significantly improve the loss of appetite in adult rats caused by lactation disorders. On days 2 and 3, the water intake of adult rats in the control group was significantly different from that in the normal group (*P* < 0.05). There was no significant difference of the water intake between the CNL group and the control group (*P* > 0.05). Furthermore, there was no significant difference in water intake between NCNLL/NCNLH groups and the control group (*P* > 0.05). These results indicated that NCNLL and NCNLH have no significant effect on the water intake of adult rats with bromocriptine-induced hypogalactia.

### 3.2. The Effect of NCNL on Serum Biochemical Parameters in Rats with Postpartum Hypogalactia

To determine the effect of NCNL on bromocriptine-induced postpartum hypogalactia in adult rats, we measured the concentration of hormones in serum. One week after administration, the serum levels of GH, PRL, DA, T3, and T4 in the control group were significantly lower than those in the normal group (*P* < 0.05). Serum T3, T4, and DA concentrations in the CNL group were significantly higher than those in the control group (*P* < 0.05). The serum concentrations of PRL and T4 in the NCNLL and NCNLH groups were significantly higher than those in the control group (*P* < 0.05). In addition, the concentrations of E2, PROG, Ca, and UREA in the control group were significantly higher than those in the normal group due to the dysfunction of lactation. The concentrations of E2, Ca, and UREA in the CNL group were significantly lower than those in the control group (*P* < 0.05). The concentrations of E2, Ca, and UREA in the NCNLL group were significantly lower than those in the control group (*P* < 0.05). The concentrations of E2 and Ca in the NCNLL group were significantly lower than those in the control group (*P* < 0.05). These results indicated that NCNLL and NCNLH can improve the bromocriptine-induced postpartum hypogalactia by regulating the level of lactation-related hormones in the serum of adult rats.

At the same time, the concentrations of INS and IGF-1 in the control group were significantly lower than those in the normal group (*P* < 0.05), while the GLU concentration in the control group was significantly higher than that in the normal group. The concentrations of INS, IGF-1, and GLU were not significantly different between the CNL group and the control group (*P* > 0.05). Interestingly, compared with the control group, the INS, IGF-1, and GLU concentrations in the NCNLL group changed significantly (*P* < 0.05). Compared with the control group, GLU concentration in the NCNLH group also changed significantly (*P* < 0.05). These results indicated that NCNLL and NCNLH can improve the disorders of glucose metabolism caused by postpartum hypogalactia in adult rats.

Finally, compared with the normal group, ALT, AST, TCHO, and TG levels in the control group were significantly increased (*P* < 0.05). After a week of intragastric administration, the levels of ALT, AST, TCHO, and TG in the CNL, NCNLL, and NCNLH groups were significantly decreased compared to those in the control group (*P* < 0.05). These results indicated that NCNLL and NCNLH can regulate the lipid metabolism disorder in adult rats with hypogalactia (Table 2).

### 3.3. The Effect of NCNL on the Pathological Alterations of the Mammary Glands in Rats with Postpartum Hypogalactia

**Figure 1:**
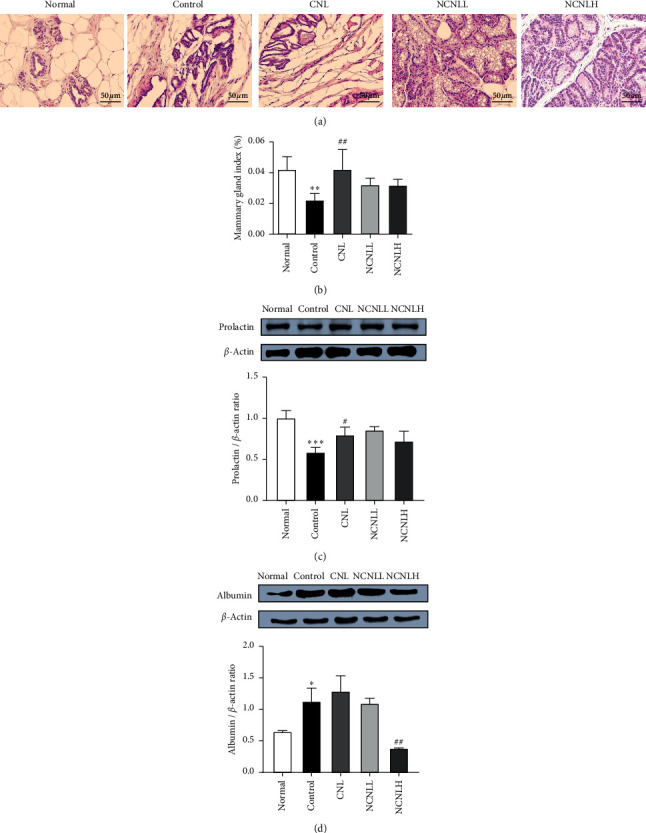
The effect of NCNL on the mammary pathology (a), mammary gland index (b), and protein expression (c, d) in rats with hypogalactia. Tissue staining with H&E (a). All tissues were observed using a light microscope; representative images are shown (×400). Mammary gland to body weight ratio (b). Expression of prolactin and albumin was determined by western blotting (c-d). Relative expression of prolactin and albumin was calculated using *β*-actin for normalization. Normal: normal group. Control: bromocriptine-induced hypogalactia control group. CNL: traditional Cui Nai Ling aqueous extract 1000 mg/kg administered group. NCNLL: new Cui Nai Ling aqueous extract 500 mg/kg administered group. NCNLH: new Cui Nai Ling aqueous extract 1000 mg/kg administered group. The data are expressed as the mean ± standard deviation (*n* = 7). ^*∗*^*P* < 0.05, ^*∗∗*^*P* < 0.01, and ^*∗∗∗*^*P* < 0.001 versus the normal group; ^#^*P* < 0.05, ^##^*P* < 0.01, and ^###^*P* < 0.001 versus the control group.

To determine the effect of NCNL on lactation disorders, we performed H&E staining for the tissues from mammary glands of the rats with postpartum hypogalactia. As shown in Figure 1(a), the mammary glands in the control group exhibited atrophy and connective tissues appeared. After drug administration, the rats in each treatment group had obvious breast acini, but there were varying degrees of acinar hyperplasia and secretion in the alveolar cavity. The mammary gland index in the control group was significantly lower than that in the normal group (*P* < 0.05). The mammary gland index in the CNL group was significantly higher than that in the control group (*P* < 0.05). However, there was no significant difference between the mammary gland index in the CNL, NCNLL, and NCNLH groups and that in the control group (*P* > 0.05) (Figure 1(b)). These results indicated that NCNLL and NCNLH can improve lactation and increase mammary gland index by improving the tissue type of breast acini in rats with hypogalactia.

### 3.4. Network Pharmacology-Based Studies on the Mechanisms of Actions of NCNL on Postpartum Hypogalactia

A total of 664 active ingredients and 5,421 targets were obtained. The lactation-related genes from the OMIM database and GeneCards database were combined, resulting in 3303 gene targets related to lactation. The ingredients and disease targets were uploaded to the online Venn diagram (http://bioinformatics.psb.ugent.be/webtools/Venn/), and a total of 165 overlapping genes associated with both active ingredients and lactation were obtained. The top 20 significant overlapping genes are presented in Table 3. The other overlapping genes are shown in Supplementary Table 1.

**Figure 2:**
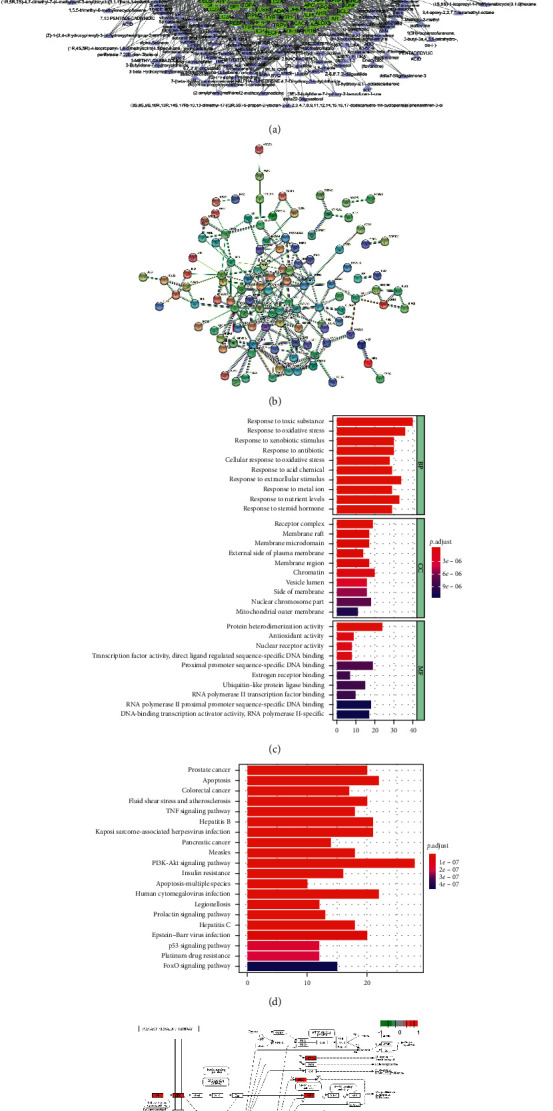
Network construction, pathway, and functional enrichment analysis of the effect of NCNL on hypogalactia. Potential active ingredient-target-disease network. (a) Different color symbols as mentioned here: disease (yellow), NCNL (red), targets (green), and compounds (purple). PPI network. (b) Node information as mentioned here: query proteins and first shell of interactors (colored nodes), the second shell of interactors (white nodes), proteins of unknown 3D structure (empty nodes), and some 3D structures being known or predicted (filled nodes), curated databases (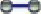), experimentally determined (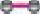), gene neighborhood (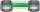), gene fusions (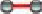), gene co-occurrence (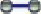), text mining (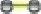), coexpression (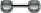), and protein homology (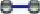). (c) GO function analysis. (d) KEGG pathway enrichment analysis: the gradual change in color represented the change in probability. (e) PI3K-AKT signaling pathway.

Cytoscape 3.7.1 (http://www.cytoscape.org/) software was used to construct the “ingredient-target-disease” interactive network between NCNL and lactation (Figure 2(a)). According to the “ingredient-target-disease” interactive network between NCNL and lactation, a total of 302 high-frequency common ingredients were obtained.

A protein-protein interaction database (STRING 11.0, https://string-db.org/) was constructed for CNL and lactation (Figure 2(b)), and the most significant 20 associated nodes in PPI are shown in Table 4. Other nodes in PPI are shown in Supplementary Table 2.

The comparative analysis resulted in a total of 30 high-frequency common targets. The central node of the PPI network between CNL and lactation was INS. In addition, protein kinase B1 (ALB), interleukin 6 (IL6), cysteine acid aspartic protease 3 (CASP3), epidermal growth factor receptor (EGFR), vascular endothelial growth factor A (VEGFA), nucleophosphoprotein (MYC), and mitogen-activated protein kinase 8 (MAPK8) also appeared frequently.

These common protein targets of CNL and lactation were uploaded to the DAVID database, and a total of 1583 GO entries and 17 high-frequency common KEGG pathways were obtained. The results were visualized using Cytoscape 3.7.1 (http://www.cytoscape.org/) (Figures 2(c) and 2(d)). The results showed that the pathways involved in NCNL and lactation included the P13K-Akt, insulin resistance, and the prolactin signaling pathway, among which the most frequent pathway was the P13K-Akt signaling pathway (Figure 2(e)).

These data provided a theoretical basis for the possibility that the antagonistic activity of NCNL against DN may be related to the activity of PI3K/Akt- and prolactin-related targets. These predicted results were verified by western blotting.

Significant inhibition of prolactin (*P* < 0.001) was observed in the control group compared with the normal group. The administration of CNL and NCLN at low and high doses significantly increased prolactin expression (Figure 1(c)).

Significant increases in albumin (*P* < 0.05) occurred in the control group compared with the normal group. However, NCNLH administration significantly decreased the expression of albumin (*P* < 0.01) (Figure 1(d)).

### 3.5. Metabolomics-Based Studies on the Mechanism of Action of NCNL on Postpartum Hypogalactia

**Figure 3:**
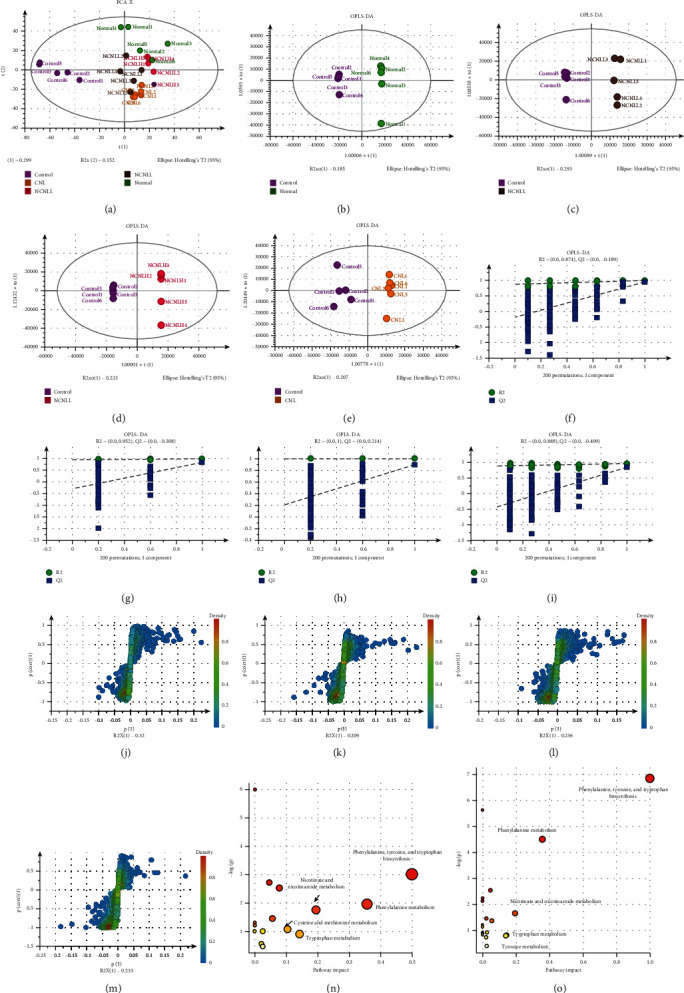
Differential metabolite analysis of serum. The PCA analysis in different groups (a) and OPLS-DA analysis between control and normal (b), NCNLL (c), NCNLH (d), and CNL (e) treatment groups. The permutation test of the OPLS-DA mode between control and normal (f), NCNLL (g), NCNLH (h), and CNL (i) treatment groups. The *S*-plot analysis between control and normal (j), NCNLL (k), NCNLH (l), and CNL (m) treatment groups. The summary of pathway analysis of differential metabolites between control and normal (n) and NCNLH (o).

Unsupervised principal component analysis (PCA) was used to analyze the metabolic profile of all samples from the female adult rats. The PCA results showed that the normal group and the control group can be completely separated, indicating that the physiological and metabolic status of the rat with bromocriptine-induced hypogalactia changed significantly. Further analysis using the OPLS-DA pattern recognition method showed that the scatter points of the normal group and the control group can be clearly separated, which is consistent with the results of PCA analysis. These results indicated that the hypogalactia model developed successfully in rats. The scatter points in CNL, NCNLL, and NCNLH groups can also be clearly distinguished from those in the control group, indicating that the overall metabolism in the drug treatment groups was significantly different from that in hypogalactia group. At the same time, the model verification results show that all *R*^2^ and *Q*^2^ values on the left were lower than the original point on the right and the intersection of the *Q*^2^ regression line and vertical axis was less than zero, indicating that the model had high reliability (Figures 3(f)–3(i)).

We performed *S*-plot (Figures 3(j)–3(m)) combined with VIP value (>3) and an independent sample *t*-test to compare the difference in metabolites between the normal and control groups and identify metabolites that were specifically associated with bromocriptine-induced hypogalactia. We obtained 30 significantly different metabolites between different groups shown in Table 5. The contents of nine metabolites including 9-oxo-10(E), 12(E)-octadecadienoic acid, abietic acid, choline, DL-carnitine, DL-stachydrine, cytosine, hypoxanthine, methyl palmitate, and trans-3-indoleacrylic acid in the control group were significantly increased compared to those in the normal group, while the contents of 21 metabolites including 2-hydroxyphenylalanine, acetyl-L-carnitine, betaine, corticosterone, creatinine, DL-tryptophan, docosahexaenoic acid, erucamide, hexadecanamide, L-phenylalanine, L-tryptophan, methionine, N,N′-diphenylguanidine, nicotinamide, oleamide, PEG n11, PEG n12, pipecolic acid, proline, spermine, and stearamide in the control group were significantly decreased compared to those in the normal group (Figure 3(j)).

Based on the metabolic profile, the NCNLH group was significantly separated from the control group, indicating that NCNLH can effectively improve bromocriptine-induced hypogalactia. Based on the level of metabolites, NCNLH can significantly reduce the concentration of 13 metabolites including 9-oxo-10(E), 12(E)-octadecadienoic acid, adenosine, choline, DL-carnitine, DL-stachydrine, DL-tryptophan, hypoxanthine, leucine, methyl palmitate, nicotinamide, oleamide, pipecolic acid, and trans-3-indoleacrylic acid. Compared with the control group, NCNLH can significantly increase the concentration of 15 metabolites including acetyl-L-carnitine, betaine, corticosterone, cytosine, docosahexaenoic acid, hexadecanamide, L-phenylalanine, L-tryptophan, L-tyrosine, N, N′-diphenylguanidine, PEG n10, PEG n11, spermine, stearamide, and taurocholic acid. NCNLH did not significantly alter the content of 2-hydroxyphenylalanine, creatinine, and PEG n14 (Figure 3(l)).

The abovementioned differential metabolites were imported into MetPA (http://www.metaboanalyst.ca/) for pathway analysis (Figures 3(n) and 3(o)). The metabolic pathway with Impact >0.1 was considered as the potential target pathway. Compared with the normal group, five metabolic pathways including phenylalanine, tyrosine, and tryptophan biosynthesis, phenylalanine metabolism, nicotinate and nicotinamide metabolism, tryptophan metabolism, and cysteine and methionine metabolism pathways were significantly altered in the control group. The metabolic pathways that were significantly upregulated by NCNLH included phenylalanine, tyrosine, and tryptophan biosynthesis, and tyrosine metabolism pathways. The pathways that were downregulated by NCNLH were cysteine and methionine metabolism pathways.

## 4. Discussion

Veterinary drugs refer to drugs and their related products used to maintain growth and development, prevent diseases, and promote production performance in livestock and poultry animals [16]. According to the report of the World Health Organization Joint Expert Committee on Food Additives (JECFA), there are approximately 120 veterinary drug residues in food. Veterinary drugs are mainly divided into antibiotics, hormones, and anthelmintics. Antibiotics are the main veterinary drug additives, accounting for 60% of the total veterinary drugs [17–19]. Extensive use of veterinary drugs not only affects the quality and safety of animal products but also damages the health of consumers due to the drug residues. Improper use of antibiotics, hormones, and chemical synthetic additives poses safety concerns to livestock products [20]. At present, there is a huge market demand for safe, effective, and low-toxic new pharmaceutical additives that are developed by traditional Chinese medicine.

Traditional Chinese medicine has a long history and has been used to prevent, diagnose, and treat diseases or regulate human physiological functions under the guidance of its unique theoretical system [21, 22]. The main source of traditional Chinese medicine is derived from plants, while a small portion is derived from animals, minerals, and biological products. There are approximately 3,000 kinds of Chinese medicines recorded in the Chinese medicine literature, and most of them were derived from wild animals and plants. They are obtained by natural processing methods to retain their natural structure and biological activities. They are used for humans and animals for a long time and have been verified to have therapeutic and health care effects [23, 24]. Under the guidance of modern analytical technology, animal nutrition, and modern engineering technology, Chinese medicine additives are prepared with the characteristics of low toxicity, few residues, low level of drug resistance, wide ranges of sources, and low prices.

In this study, we added Tetrapanacis Medulla and replaced Vaccariae Semen with fried Vaccariae Semen in CNL described in the 2015 edition of “Chinese Veterinary Pharmacopoeia”. Vaccariae Semen has been used to reduce swelling for breast carbuncle or other types of sore carbuncle and pain [25]. Frying can easily produce the effective ingredients of Vaccariae Semen with warm characteristics, strong dispersing effect, better ability to promoter blood circulation, menstruation, and lactation. Fried Vaccariae Semen is mostly used for postpartum milk retention. Zhou et al. [26, 27] demonstrated that the dissolution rate of cyclic peptides, erythronine, and flavonoid glycosides in Vaccariae Semen increased significantly in water decoction after frying. *Tetrapanax papyrifer* is sweet, light, and slightly cold and is mostly used for edema and postpartum milk retention. Tetrapanacis Medulla can promote the stomach gas to move up and increase lactation [28]. Combined with Chuanxiong Rhizoma in CNL, it enhances the function of gas regulation and promotes blood circulation and lactation. Through the above-described ingredient adjustments, the effect of NCNL on dysmenorrhea and lactation is enhanced. The results of these studies showed that NCNLL and NCNLH can significantly improve the loss of appetite in female adult rats and significantly reduce the weight loss of pups due to lactation disorders in adults.

The synthesis of milk and lactation is a complex physiological process controlled by the neuroendocrine system, including a variety of endocrine hormones [29]. It is well recognized that PRL secreted by the pituitary gland is the most important hormone in the process of lactation [30]. RPL plays an important role in the maintenance of lactation. Various other factors regulate milk synthesis and lactation through prolactin. Therefore, the occurrence of postpartum hypogalactia is the process and consequence of the decreased synthesis and secretion of breast milk due to the reduced secretion of prolactin by the anterior pituitary gland or inhibition of its function. The secretion of prolactin is mainly regulated by the neuroendocrine system [31]. For example, secretion of prolactin by the prolactin-secreting cells in the pituitary gland is regulated by the signal balance between the secreted inhibitors and stimulators from the hypothalamus as well as the hormones in the peripheral blood. Among them, the prolactin inhibitory factor (PIF) secreted by the hypothalamus includes DA and r-aminobutyric acid, while the prolactin-releasing factor (PRF) includes gonadotropin-releasing hormone, thyrotropin-releasing hormone, vasoactive intestinal polypeptide, and angiotensin II. In addition, neurotransmitters in the brain, e.g., catechins and serotonin, can also promote the release of prolactin. Various internal and external factors, such as nutritional deficiency and stress, can increase the level of PIF through the neuroendocrine system. When PRF decreases, secretion of prolactin is reduced, resulting in a decrease in the concentration of prolactin in the blood and decreases or termination of milk secretion and postpartum hypogalactia or agalactia [29]. In this study, compared with the control group, the NCNLL and NCNLH groups can regulate the levels of PRL, T4, E2, Ca, UREA, GLU, ALT, AST, TCHO, and TG in the serum, indicating that NCNL at either low or high doses can improve bromocriptine-induced lactation disorder, glucose, and lipid metabolism disorder through regulating the level of lactation-associated hormones in the serum of female adult rats. Such mechanisms were further explored and verified by network pharmacology studies. INS is the core target of NCNL in bromocriptine-induced hypogalactia. INS-associated pathways include the P13K-Akt signaling, insulin resistance, and prolactin signaling pathways among which the most frequent pathway is the PI3K-Akt signaling pathway. There are INS and its receptors in breast tissue; thus, INS can directly act on the breast through the receptor and have a regulatory effect on the final differentiation, maturity, and milk production of the breast, as well as the growth and development of newborns. NCNL can promote the proliferation of mammary gland cells through the PI3K-Akt signaling pathway, regulate the level of insulin through the insulin resistance signaling pathway, and increase lactation through the prolactin signaling pathway.

In addition, the metabolism of mammary gland nutrients is also an important factor affecting lactation [32]. Whey protein is the most important nutrient in milk and one of the important indicators to measure milk quality [33]. As the basic unit of whey protein synthesis, amino acids are actively metabolized in the mammary gland and are closely related to lactation [34]. Most free amino acids adsorbed from blood to the mammary glands are used for whey protein synthesis. Amino acids in the mammary glands also play an important role in metabolism regulation. Amino acids enter the mammary epithelial cells through the transporter and undergo protein synthesis and degradation processes. The metabolism of amino acids in the mammary gland is complex, and the metabolic process and efficiency of various amino acid metabolism need to be further revealed. In this study, the metabolomics results showed that the metabolic pathways that are different between control and normal groups or between control and each treatment group were concentrated in amino acid metabolism. For example, compared to the normal group, five metabolic pathways including phenylalanine, tyrosine, and tryptophan biosynthesis, phenylalanine metabolism, nicotinate and nicotinamide metabolism, tryptophan metabolism, and cysteine and methionine metabolism pathways were altered significantly in the control group. Compared to the control group, phenylalanine, tyrosine, and tryptophan biosynthesis, and tyrosine metabolism pathways were upregulated, while cysteine and methionine metabolism pathways were downregulated in the NCNLH group. These results suggested that NCNL can increase the uptake of essential amino acids by the mammary gland and the balanced uptake of nonessential amino acids, which ultimately improves the bromocriptine-induced postpartum hypogalactia.

To the best of our knowledge, this is the first study using metabolomics coupled with network pharmacology to reveal the mechanisms underlying the therapeutic effect of an NCNL on bromocriptine-induced hypogalactia. However, we performed metabolic profiling only by nontargeted metabolomics. Future studies will focus on validation of the results by targeted metabolomics and improving the specificity enriched by network pharmacology and association with target diseases.

## 5. Conclusions

In this study, we modified CNL by adding or replacing the original component to achieve NCNL and applied NCNL in the bromocriptine-induced postpartum hypogalactia model. Our results demonstrated that NCNL can significantly increase the serum prolactin concentration in rats with bromocriptine-induced postpartum hypogalactia. Furthermore, NCNL can significantly improve the glucose and lipid metabolism disorders and affect amino acid metabolism through PI3K-Akt, insulin resistance, and prolactin pathways to achieve its therapeutic effect. These findings revealed the effect and application value of NCNL on postpartum hypogalactia in animals. However, the safety of the milk and the growth and metabolism parameters in the pups require further evaluation in future studies.

## Figures and Tables

**Table 1:** Effect of NCNL on the physiological signs of rats with postpartum hypogalactia.

	Group	D0	D1	D2	D3	D4	D5	D6	D7
*Rat weight (g)*	Normal	316.00 ± 26.67	318.25 ± 27.13	314.00 ± 26.18	319.00 ± 25.43	320.00 ± 28.22	312.75 ± 25.76	320.50 ± 27.89	323.50 ± 28.34
	Control	310.80 ± 21.33	311.80 ± 23.61	293.00 ± 25.88	317.00 ± 24.89	311.60 ± 24.95	314.20 ± 23.64	315.60 ± 22.90	317.20 ± 21.35
	CNL	307.00 ± 14.76	315.00 ± 11.45	287.57 ± 9.39	305.14 ± 8.59	302.57 ± 8.66	304.85 ± 8.49	305.00 ± 8.16	305.43 ± 10.98
	NCNLL	309.00 ± 11.36	319.00 ± 16.70	288.20 ± 16.03	310.60 ± 11.51	309.60 ± 14.58	308.40 ± 16.64	310.00 ± 18.70	309.40 ± 17.49
	NCNLH	314.66 ± 25.75	319.67 ± 37.75	299.67 ± 29.89	318.67 ± 28.50	316.16 ± 27.72	318.16 ± 29.05	316.67 ± 28.93	313.50 ± 25.19

*Pups' weight (g)*	Normal	112.74 ± 22.35	132.62 ± 22.11	152.82 ± 22.64	173.66 ± 22.38	194.96 ± 21.79	216.40 ± 21.90	236.66 ± 21.98	259.72 ± 21.36
	Control	109.65 ± 6.20	115.88 ± 5.44	120.53 ± 4.89^*∗*^	128.00 ± 6.59^*∗*^	131.83 ± 7.56^*∗*^	133.58 ± 6.99^*∗*^	130.48 ± 6.42^*∗*^	129.30 ± 5.81^*∗*^
	CNL	103.07 ± 5.62	110.46 ± 5.16	117.77 ± 5.35	125.13 ± 8.21	133.39 ± 10.78	140.14 ± 13.77	145.83 ± 18.05	153.41 ± 22.62^#^
	NCNLL	109.06 ± 6.13	119.96 ± 9.55	128.04 ± 10.74	140.70 ± 13.59	152.02 ± 16.19	163.52 ± 18.83^#^	172.98 ± 20.58^#^	188.10 ± 23.23^#^
	NCNLH	107.08 ± 7.42	112.65 ± 10.71	123.87 ± 10.30	133.50 ± 13.38	142.95 ± 16.46	151.45 ± 19.76	157.52 ± 23.86^#^	171.05 ± 26.89^#^

*Food intake (g)*	Normal	35.23 ± 2.91	32.66 ± 2.31	39.05 ± 5.82	42.29 ± 4.28	45.12 ± 3.01	46.56 ± 6.09	48.16 ± 6.45	49.88 ± 2.17
	Control	26.62 ± 8.55^*∗*^	29.15 ± 7.03^*∗*^	29.35 ± 4.69^*∗*^	38.33 ± 4.02^*∗*^	32.14 ± 5.33^*∗*^	28.44 ± 4.47^*∗*^	27.99 ± 1.57^*∗*^	27.59 ± 4.43^*∗*^
	CNL	26.92 ± 2.43	31.88 ± 4.40	23.21 ± 3.02	31.11 ± 6.77	31.22 ± 3.90	31.29 ± 5.62	30.72 ± 6.06	30.52 ± 5.69
	NCNLL	30.19 ± 7.77	36.95 ± 4.82	30.75 ± 7.71	37.69 ± 4.76	37.26 ± 4.54	40.94 ± 6.43^#^	37.39 ± 5.46^#^	37.30 ± 4.20^#^
	NCNLH	30.04 ± 11.57	32.99 ± 6.93	26.59 ± 4.31	36.68 ± 3.93	35.56 ± 6.09	37.18 ± 5.68^#^	34.18 ± 3.85^#^	35.90 ± 4.05^#^

*Water intake (mL)*	Normal	49.38 ± 4.80	50.37 ± 10.54	56.13 ± 7.63	67.90 ± 16.33	62.46 ± 12.63	59.60 ± 15.58	60.79 ± 14.84	67.81 ± 13.00
	Control	57.74 ± 13.95	65.16 ± 9.29	24.74 ± 6.32^*∗*^	91.20 ± 16.54^*∗*^	67.40 ± 8.39	60.24 ± 19.39	56.35 ± 14.54	54.01 ± 11.64
	CNL	53.97 ± 7.92	61.25 ± 8.94	16.93 ± 3.85	81.69 ± 23.89	59.92 ± 10.93	58.73 ± 12.35	56.00 ± 11.51	60.93 ± 13.71
	NCNLL	59.98 ± 19.39	54.60 ± 9.72	24.65 ± 7.63	80.79 ± 31.63	59.83 ± 14.79	58.95 ± 16.74	62.46 ± 14.68	63.37 ± 13.93
	NCNLH	66.53 ± 27.68	71.27 ± 23.85	26.12 ± 7.96	96.75 ± 25.53	71.22 ± 28.43	71.76 ± 20.98	79.22 ± 32.22	76.98 ± 27.13

The data are expressed as the mean ± SD (*n* = 7); ^*∗*^*P* < 0.05 versus the normal group; ^#^*P* < 0.05 versus the control group. Normal: normal group. Control: bromocriptine-induced hypogalactia control group. CNL: traditional Cui Nai Ling aqueous extract 1000 mg/kg administered group. NCNLL: new Cui Nai Ling aqueous extract 500 mg/kg administered group. NCNLH: new Cui Nai Ling aqueous extract 1000 mg/kg administered group.

**Table 2:** The effect of NCNL on serum biochemical parameters in rats with postpartum hypogalactia.

	Normal	Control	CNL	NCNLL	NCNLH
GH (ng/mL)	20.61 ± 1.97	17.51 ± 0.63^*∗*^	16.69 ± 1.44	15.48 ± 1.62	16.37 ± 1.07
PRL (ng/L)	160.33 ± 10.43	121.61 ± 2.61^*∗*^	130.33 ± 13.01	155.38 ± 9.28^#^	134.26 ± 8.55
DA (ng/L)	16.42 ± 0.63	13.31 ± 1.43^*∗*^	16.84 ± 2.06^#^	15.52 ± 0.45	16.08 ± 1.64
T3 (ng/mL)	9.49 ± 0.54	7.95 ± 0.24^*∗*^	9.33 ± 0.74^#^	9.01 ± 0.53	7.99 ± 0.41
T4 (ng/mL)	214.02 ± 23.76	142.10 ± 15.51^*∗*^	209.25 ± 20.49^#^	190.14 ± 10.35^#^	177.81 ± 18.17^#^
E2 (pg/mL)	175.35 ± 17.14	226.23 ± 13.16^*∗*^	197.40 ± 6.85^#^	199.81 ± 6.32^#^	187.74 ± 2.39^#^
PROG (ng/mL)	12.98 ± 0.28	14.45 ± 0.25^*∗*^	13.18 ± 0.31^#^	13.84 ± 0.03	14.18 ± 0.24
Ca (pg/mL)	2.09 ± 0.08	2.46 ± 0.13^*∗*^	2.15 ± 0.11	1.89 ± 0.14^#^	2.07 ± 0.11^#^
UREA (U/L)	7.66 ± 0.77	9.30 ± 0.89^*∗*^	7.15 ± 0.37^#^	6.76 ± 0.69^#^	7.91 ± 1.05
INS (mU/L)	45.19 ± 3.32	37.69 ± 1.99^*∗*^	38.94 ± 5.42	47.89 ± 4.24^#^	39.76 ± 3.11
IGF-1 (ng/mL)	26.98 ± 0.26	23.76 ± 0.84^*∗*^	26.24 ± 1.45	27.62 ± 2.84^#^	24.75 ± 2.92
GLU (mg/dL)	7.62 ± 0.98	10.45 ± 1.25^*∗*^	8.31 ± 0.60	6.60 ± 1.02^#^	7.09 ± 0.80^#^
ALT (U/L)	26.00 ± 3.16	32.00 ± 3.58^*∗*^	18.50 ± 1.66^#^	12.50 ± 0.87^#^	15.50 ± 1.66^#^
AST (U/L)	148.00 ± 21.23	195.25 ± 14.31^*∗*^	144.00 ± 9.09^#^	122.00 ± 14.24^#^	144.67 ± 7.36^#^
TCHO (mmol/L)	1.56 ± 0.11	1.95 ± 0.28^*∗*^	1.69 ± 0.14^#^	1.68 ± 0.14^#^	1.51 ± 0.17^#^
TG (mmol/L)	0.53 ± 0.10	0.83 ± 0.09^*∗*^	0.66 ± 0.07^#^	0.60 ± 0.09^#^	0.42 ± 0.02^#^

The data are expressed as the mean ± SD (*n* = 7); ^*∗*^*P* < 0.05 versus the normal group; ^#^*P* < 0.05 versus the control group. Normal: normal group. Control: bromocriptine-induced hypogalactia control group. CNL: traditional Cui Nai Ling aqueous extract 1000 mg/kg administered group. NCNLL: new Cui Nai Ling aqueous extract 500 mg/kg administered group. NCNLH: new Cui Nai Ling aqueous extract 1000 mg/kg administered group.

**Table 3:** The top 20 significant overlapping genes between NCNL and lactation.

No.	Overlapping genes
1	Prostaglandin-endoperoxide synthase 1 (PTGS1)
2	Coagulation factor VII (F7)
3	Gamma-aminobutyric acid type A receptor subunit alpha-1 (GABRA1)
4	Transient receptor potential cation channel subfamily V member 1 (TRPV1)
5	Cholinergic receptor muscarinic 2 (CHRM2)
6	RELA proto-oncogene, NF-KB subunit (RELA)
7	Proliferating cell nuclear antigen (PCNA)
8	Albumin (ALB)
9	MYC proto-oncogene, BHLH transcription factor (MYC)
10	Uncoupling protein 2 (UCP2)
11	Progesterone receptor (PGR)
12	Nuclear receptor subfamily 3 group C member 2 (NR3C2)
13	Nuclear receptor coactivator 1 (NCOA1)
14	Aldo-keto reductase family 1 member B (AKR1B1)
15	Plasminogen activator, urokinase (PLAU)
16	Alcohol dehydrogenase 1B (class I), beta polypeptide (ADH1B)
17	Telomerase-associated protein 1 (TEP1)
18	Erb-b2 receptor tyrosine kinase 2 (ERBB2)
19	Peroxisome proliferator-activated receptor gamma (PPARG)
20	Lipoprotein lipase (LPL)

**Table 4:** The value for the most significant 20 nodes in the PPI network between NCNL and lactation.

Node value	Proteins
102	Insulin (INS)
98	Albumin (ALB)
90	Interleukin 6 (IL6)
84	Caspase 3 (CASP3)
80	Epidermal growth factor receptor (EGFR)
79	Vascular endothelial growth factor A (VEGFA)
78	MYC proto-oncogene (MYC)
76	Mitogen-activated protein kinase 8 (MAPK8)
65	Estrogen receptor 1 (ESR1)
64	Cyclin D1 (CCND1)
60	Cytochrome c (CYCS)
60	Fos proto-oncogene (FOS)
59	Peroxisome proliferator-activated receptor gamma (PPARG)
52	Androgen receptor (AR)
52	Erb-b2 receptor tyrosine kinase 2 (ERBB2)
51	RELA proto-oncogene (RELA)
48	Catenin beta-1 (CTNNB1)
46	Caspase 8 (CASP8)
45	MDM2 proto-oncogene (MDM2)
44	Apolipoprotein B (APOB)

Node value: the higher the degree value, the closer the interaction.

**Table 5:** Comparison of metabolites between different groups.

No.	Differential metabolites	Formula	Normal vs. control up-down	Control vs. NCNLH up-down
1	9-Oxo-10(E)	C_18_ H_30_ O_3_	—	Up
2	12(E)-Octadecadienoic acid	C_18_ H_30_ O_3_	—	Up
3	Abietic acid	C_20_ H_30_ O_2_	—	Up
4	Choline	C_5_ H_13_ N O	Down	Up
5	DL-Carnitine	C_7_ H_15_ N O_3_	—	—
6	DL-Stachydrine	C_7_ H_13_ N O_2_	Up	—
7	Cytosine	C_4_ H_5_ N_3_ O	Up	—
8	Hypoxanthine	C_5_ H_4_ N_4_ O	UP	Up
9	Methyl palmitate	C_17_ H_34_ O_2_	—	Up
10	*trans*-3-Indoleacrylic acid	C_11_ H_9_ N O_2_	Up	Up
11	2-Hydroxyphenylalanine	C_9_ H_11_ N O_3_	—	—
12	Acetyl-L-carnitine	C_9_ H_17_ N O_4_	Up	Up
13	Betaine	C_5_ H_11_ N O_2_	—	Up
14	Corticosterone	C_21_ H_30_ O_4_	Up	—
15	Creatinine	C_4_ H_7_ N_3_ O	—	Up
16	DL-Tryptophan	C_11_ H_12_ N_2_ O_2_	Up	Up
17	Docosahexaenoic acid	C_22_ H_32_ O2	Up	Up
18	Erucamide	C_22_ H_43_ N O	Up	—
19	Hexadecanamide	C_16_ H_33_ N O	—	Up
20	L-Phenylalanine	C_9_ H_11_ N O_2_	—	Up
21	L-Tryptophan	C_11_ H_12_ N_2_ O_2_	Up	Up
22	Metalionine	C_5_ H_11_ N O_2_ S	Down	—
23	N,N′-Diphenylguanidine	C_13_ H_13_ N_3_	Up	
24	Oleamide	C_18_ H_35_ N O	—	Up
25	PEG n11	C_22_ H_46_ O_12_	Up	—
26	PEG n12	C_24_ H_50_ O_13_	Down	—
27	Pipecolic acid	C_6_ H_11_ N O_2_	Up	Up
28	Proline	C_5_ H_9_ N O_2_	—	—
29	Spermine	C_10_ H_26_ N_4_	—	Up
30	Stearamide	C_18_ H_37_ N O	Up	Up

Normal: normal group. Control: bromocriptine-induced hypogalactia control group. NCNLH: new Cui Nai Ling aqueous extract 1000 mg/kg administered group.

## Data Availability

The data used to support the results of this study are available from the corresponding author upon request.

## Abbreviations

CNL: Traditional Cui Nai Ling treatment group
NCNLL: New CNL low-dose treatment group
NCNLH: NCNL high-dose treatment group
PRL: Prolactin
PROG: Progesterone
E2: Estradiol
T3: Triiodothyronine
T4: Thyroxine
GH: Growth hormone
INS: Insulin
IGF-1: Insulin-like growth factor-1
DA: Dopamine
ALT: Alanine aminotransferase
AST: Aspartate aminotransferase
GLU: Blood glucose
TCHO: Total cholesterol
TG: Triglycerides
Ca: Blood calcium
UREA: Urea
OPLS-DA: Orthogonal partial least squares discriminant analysis
OB: Bioavailability
DL: Drug-like properties
PPI: Protein-protein interaction
SD: Standard deviation
ALB: Protein kinase B1
IL6: Interleukin 6
CASP3: Caspase 3
EGFR: Epidermal growth factor receptor
VEGFA: Vascular endothelial growth factor A
MYC: Nucleophosphoprotein
MAPK8: Mitogen-activated protein kinase 8
JECFA: World Health Organization Joint Expert Committee on Food Additives
PIF: Prolactin inhibitor
PRF: Prolactin release factor.

## Supplementary Materials

Supplementary Materials Supplementary Table 1: overlapping genes between NCNL and lactation. Supplementary Table 2: node values in the protein-protein interaction network between NCNL and lactation.
