# Supplementary material for: Metabolomics and Network Pharmacology-Based Investigation into the Mechanisms Underlying the Therapeutic Effect of a New Chinese Traditional Medicine (Cui Nai Ling) on Bromocriptine-Induced Hypogalactia
Source: Evid Based Complement Alternat Med. 2021 Jun 16;2021:8857449. doi: 10.1155/2021/8857449 (PMC8221871; doi:10.1155/2021/8857449)
Supplement: Supplementary Materials — Supplementary Table 1: overlapping genes between NCNL and lactation. Supplementary Table 2: node values in the protein-protein interaction network between NCNL and lactation. [file 8857449.f1.docx]

**Supplementary Table 1 Overlapping genes between NCNL and lactation**

| **NO.** | **Overlapping genes** | **NO.** | **Overlapping genes** | **NO.** | **Overlapping genes** | **NO.** | **Overlapping genes** |
| --- | --- | --- | --- | --- | --- | --- | --- |
| 1 | Prostaglandin-endoperoxide synthase 1（PTGS1） | 43 | Fos Proto-Oncogene, AP-1 Transcription Factor Subunit (FOS) | 85 | Glutamate ionotropic receptor AMPA type subunit 2 (GRIA2) | 127 | Gephyrin (GPHN) |
| 2 | Coagulation factor VII (F7) | 44 | Eukaryotic Translation Initiation Factor 6 (EIF6) | 86 | Oxidized low density lipoprotein receptor 1 (OLR1) | 128 | Aldehyde dehydrogenase 5 family member A1 (ALDH5A1) |
| 3 | Gamma-Aminobutyric Acid Type A Receptor Subunit Alpha1 (GABRA1) | 45 | Caspase 9(CASP9) | 87 | Cell division cycle 25C (CDC25C) | 129 | Ornithine transcarbamylase (OTC) |
| 4 | Transient Receptor Potential Cation Channel Subfamily V Member 1 (TRPV1) | 46 | RB Transcriptional Corepressor 1 (RB1) | 88 | Apoptotic peptidase activating factor 1 (APAF1) | 130 | Succinate dehydrogenase complex flavoprotein subunit A (SDHA) |
| 5 | Cholinergic receptor muscarinic 2 (CHRM2) | 47 | Interleukin 6 (IL6) | 89 | Polo like kinase 1 (PLK1) | 131 | Solute carrier family 25 member 10 (SLC25A10) |
| 6 | RELA Proto-Oncogene, NF-KB Subunit (RELA) | 48 | Caspase 3 (CASP3) | 90 | Carnitine O-acetyltransferase (CRAT) | 132 | Dopamine receptor D2 (DRD2) |
| 7 | Proliferating Cell Nuclear Antigen (PCNA) | 49 | NFKB Inhibitor Alpha (NFKBIA) | 91 | 5-Hydroxytryptamine receptor 3A (HTR3A) | 133 | Lecithin-cholesterol acyltransferase (LCAT) |
| 8 | Albumin (ALB) | 50 | Cytochrome P450 Oxidoreductase (POR) | 92 | Glycine amidino transferase (GATM) | 134 | JunB proto-oncogene, AP-1 transcription factor subunit (JUNB) |
| 9 | MYC Proto-Oncogene, BHLH Transcription Factor (MYC) | 51 | Caspase 8 (CASP8) | 93 | Solute carrier family 6 member 1 (SLC6A1) | 135 | Transforming growth factor alpha (TGFA) |
| 10 | Uncoupling protein2 (UCP2) | 52 | Raf-1 proto-oncogene, serine/threonine kinase (RAF1) | 94 | Low density lipoprotein receptor (LDLR) | 136 | Kallikrein related peptidase 3 (KLK3) |
| 11 | Progesterone Receptor (PGR) | 53 | Protein kinase C alpha (PRKCA) | 95 | Microsomal triglyceride transfer protein (MTTP) | 137 | Hemoglobin subunit beta (HBB) |
| 12 | Nuclear receptor subfamily 3 group C member 2 (NR3C2) | 54 | Hypoxia inducible factor 1 subunit alpha (HIF1A) | 96 | Apolipoprotein B (APOB) | 138 | Spi-1 proto-oncogene (SPI1) |
| 13 | Nuclear receptor coactivator 1 (NCOA1) | 55 | Acetyl-CoA Carboxylase Alpha (ACACA) | 97 | Superoxide dismutase 2 (SOD2) | 139 | MDM2 proto-oncogene (MDM2) |
| 14 | Aldo-keto reductase family 1 member B (AKR1B1) | 56 | Cytochrome P450 family 3 subfamily A member 4 (CYP3A4) | 98 | Carnitine palmitoyl transferase 1A (CPT1A) | 140 | Amyloid beta precursor protein (APP) |
| 15 | Plasminogen activator, urokinase (PLAU) | 57 | Caveolin 1 (CAV1) | 99 | Ras homolog family member A (RHOA) | 141 | Caspase 7 (CASP7) |
| 16 | Alcohol dehydrogenase 1B (class I), beta polypeptide (ADH1B) | 58 | Cytochrome P450 family 1 subfamily A member 1 (CYP1A1) | 100 | Fragment of IgE receptor II (FCER2) | 142 | Tyrosinase (TYR) |
| 17 | Telomerase-associated protein 1 (TEP1) | 59 | Intercellular adhesion molecule 1 (ICAM1) | 101 | Transient Receptor Potential Cation Channel Subfamily M Member 2 (TRPM2) | 143 | Inhibitor of nuclear factor kappa B kinase regulatory subunit gamma (IKBKG) |
| 18 | Erb-b2 receptor tyrosine kinase 2 (ERBB2) | 60 | Selectin E (SELE) | 102 | Beta-1,4-Galactosyltransferase 4(B4GALT4) | 144 | Cytochrome c, somatic (CYCS) |
| 19 | Peroxisome proliferator activated receptor gamma (PPARG) | 61 | Vascular cell adhesion molecule 1 (VCAM1) | 103 | Growth Hormone Receptor (GHR) | 145 | CASP8 and FADD like apoptosis regulator (CFLAR) |
| 20 | Lipoprotein Lipase (LPL) | 62 | Baculoviral IAP repeat containing 5 (BIRC5) | 104 | Prolyl 4-hydroxylase subunit beta (P4HB) | 146 | Interleukin 13 (IL13) |
| 21 | Brain-derived neurotrophic factor (BDNF) | 63 | Nitric oxide synthase 3 (NOS3) | 105 | Breast Associated Protein-1 (BAP1) | 147 | Alkaline phosphatase, intestinal (ALPI) |
| 22 | C-reactive protein (CRP) | 64 | Heat Shock Protein Family B (Small) Member 1 (HSPB1) | 106 | RAD51 recombinase (RAD51) | 148 | Glucose-6-phosphatase, catalytic (G6PC) |
| 23 | Paraoxonase-1 (PON1) | 65 | Maltase-glucoamylase (MGAM) | 107 | Integrin subunit beta 2 (ITGB2) | 149 | APC Regulator of WNT Signaling Pathway (APC) |
| 24 | Insulin (INS) | 66 | Cyclin B1 (CCNB1) | 108 | Thromboxane A2 receptor (TBXA2R) | 150 | Tubulin beta 1 class VI (TUBB1) |
| 25 | Fatty Acid Binding Protein 1 (FABP1) | 67 | Arachidonate 5-lipoxygenase (ALOX5) | 109 | Haptoglobin (HP) | 151 | Solute carrier family 7 member 7 (SLC7A7) |
| 26 | Solute carrier family 2 member 2 (SLC2A2) | 68 | Glutathione S-transferase pi 1 (GSTP1) | 110 | Peroxisome proliferator activated receptor gamma coactivator 1 alpha (PPARGC1A) | 152 | Adenosine deaminase (ADA) |
| 27 | Peptidyl glycine alpha-amidating monooxygenase (PAM) | 69 | Nuclear factor, erythroid 2 like 2 (NFE2L2) | 111 | Mitogen-activated protein kinase 8 (MAPK8) | 153 | Choline acetyltransferase (CHAT) |
| 28 | Cathepsin D (CTSD) | 70 | NAD(P)H quinone dehydrogenase 1 (NQO1) | 112 | Glutamic-oxaloacetic transaminase 2 (GOT2) | 154 | Mitogen-activated protein kinase 10 (MAPK10) |
| 29 | Rhodopsin (RHO) | 71 | Poly (ADP-ribose) polymerase 1 (PARP1) | 113 | Malic enzyme 2 (ME2) | 155 | Abl interactor 1 (ABI1) |
| 30 | BCL2 Apoptosis Regulator (BCL2) | 72 | Aryl hydrocarbon receptor (AHR) | 114 | Aldo-keto reductase family 1 member C1 (AKR1C1) | 156 | Sterol regulatory element binding transcription factor 2 (SREBF2) |
| 31 | Solute carrier family 22 member 5 (SLC22A5) | 73 | Solute Carrier Family 2 Member 4 (SLC2A4) | 115 | Cytochrome P450 family 2 subfamily A member 6 (CYP2A6) | 157 | Sterol regulatory element binding transcription factor 1 (SREBF1) |
| 32 | Estrogen receptor 1 (ESR1) | 74 | Checkpoint kinase 2 (CHEK2) | 116 | Peptidylprolyl isomerase A (PPIA) | 158 | Sucrase-isomaltase (SI) |
| 33 | Androgen receptor (AR) | 75 | Heat shock transcription factor 1 (HSF1) | 117 | D-amino acid oxidase (DAO) | 159 | Hexokinase 1 (HK1) |
| 34 | Estrogen receptor 2 (ESR2) | 76 | Runt related transcription factor 2 (RUNX2) | 118 | 2 Receptor subunit alpha (2RA) | 160 | Cathepsin B (CTSB) |
| 35 | Glycogen synthase kinase 3 beta (GSK3B) | 77 | Ras association domain family member 1 (RASSF1) | 119 | Caspase 2 (CASP2) | 161 | Fatty acid synthase (FASN) |
| 36 | Inhibitor of nuclear factor kappa B kinase subunit beta (IKBKB) | 78 | Insulin like growth factor binding protein 3 (IGFBP3) | 120 | Catenin beta 1 (CTNNB1) | 162 | Activating transcription factor 2 (ATF2) |
| 37 | Sp1 transcription factor (SP1) | 79 | Insulin like growth factor 2 (IGF2) | 121 | MCL1 Apoptosis Regulator, BCL2 Family Member (MCL1) | 163 | Protein tyrosine phosphatase non-receptor type 6 (PTPN6) |
| 38 | Acetylcholinesterase (Cartwright Blood Group) (ACHE) | 80 | Interferon regulatory factor 1 (IRF1) | 122 | Cyclin D2 (CCND2) | 164 | Caspase 1 (CASP1) |
| 39 | Nuclear receptor subfamily 3 group C member 1 (NR3C1) | 81 | Erb-b2 receptor tyrosine kinase 3 (ERBB3) | 123 | CD36 molecule (CD36) | 165 | Microsomal glutathione S-transferase 1 (MGST1) |
| 40 | Epidermal growth factor receptor (EGFR) | 82 | Iodothyronine deiodinase 1 (DIO1) | 124 | Glial fibrillary acidic protein (GFAP) | — | — |
| 41 | Vascular endothelial growth factor A (VEGFA) | 83 | Hexokinase 2 (HK2) | 125 | Selectin P (SELP) | — | — |
| 42 | Cyclin D1 (CCND1) | 84 | Glutathione S-transferase mu 1 (GSTM1) | 126 | Solute carrier family 36 member 1 (SLC36A1) | — | — |

**Supplementary Table 2 Nodes values in the protein-protein interaction network between NCNL and lactation**

| **Proteins** | **Node value** | **Protein** | **Node value** | **Protein** | **Node value** |
| --- | --- | --- | --- | --- | --- |
| Insulin (INS) | 102 | Interleukin 13(IL13) | 27 | Proliferating cell nuclear antigen (PCNA) | 15 |
| Albumin (ALB) | 98 | Protein kinase C alpha (PRKCA) | 27 | Selectin P (SELP) | 15 |
| Interleukin 6(IL6) | 90 | Nuclear receptor coactivator (1NCOA1) | 26 | Solute carrier family 2 member 2(SLC2A2) | 15 |
| Caspase 3(CASP3) | 84 | Plasminogen activator, urokinase (PLAU) | 26 | Activating transcription factor 2(ATF2) | 14 |
| epidermal growth factor receptor (EGFR) | 80 | Uncoupling protein 2(UCP20 | 26 | JunB proto-oncogene (JUNB) | 14 |
| vascular endothelial growth factor A (VEGFA) | 79 | Carnitine palmitoyltransferase 1A (CPT1A) | 25 | Arachidonate 5-lipoxygenase (ALOX5) | 13 |
| MYC proto-oncogene (MYC) | 78 | Cytochrome P450 family 3 subfamily A member 4(CYP3A4) | 25 | Glutathione S-transferase mu 1(GSTM1) | 13 |
| mitogen-activated protein kinase 8(MAPK8) | 76 | Caspase 7(CASP7) | 24 | Protein tyrosine phosphatase non-receptor type 6(PTPN6) | 13 |
| estrogen receptor 1(ESR1) | 65 | CASP8 and FADD like apoptosis regulator (CFLAR) | 24 | Orthologs from vertebrates CHAT | 12 |
| cyclin D1(CCND1) | 64 | Cathepsin D (CTSD) | 24 | Interleukin 2 receptor subunit alpha (IL2RA) | 12 |
| cytochrome c(CYCS) | 60 | Glucose-6-phosphatase, catalytic (G6PC) | 24 | Lecithin-cholesterol acyltransferase (LCAT) | 12 |
| Fos proto-oncogene (FOS) | 60 | Insulin like growth factor binding protein 3(IGFBP3) | 24 | Cytochrome P450 family 2 subfamily A member 6(CYP2A6) | 11 |
| Peroxisome proliferator activated receptor gamma (PPARG) | 59 | Glutathione S-transferase pi 1(GSTP1) | 23 | Nuclear receptor subfamily 3 group C member 2(NR3C2) | 11 |
| Androgen receptor (AR) | 52 | polo like kinase 1(PLK1) | 23 | Prostaglandin-endoperoxide synthase 1(PTGS1) | 11 |
| Erb-b2 receptor tyrosine kinase 2(ERBB2) | 52 | RUNX family transcription factor 2(RUNX2) | 23 | Transforming growth factor alpha (TGFA) | 11 |
| RELA proto-oncogene (RELA) | 51 | apoptotic peptidase activating factor 1(APAF1) | 22 | ADA orthologs from vertebrates (ADA) | 10 |
| Catenin beta 1(CTNNB1) | 48 | CD36 molecule (CD360) | 22 | Ornithine transcarbamylase (OTC) | 10 |
| Caspase 8(CASP8) | 46 | Glutamic-oxaloacetic transaminase 2(GOT2) | 22 | Tyrosinase (TYR) | 10 |
| MDM2 proto-oncogene (MDM2) | 45 | NAD(P)H quinone dehydrogenase 1(NQO1) | 22 | Peptidylprolyl isomerase A (PPIA) | 9 |
| Apolipoprotein B(APOB) | 44 | Acetyl-CoA carboxylase alpha (ACACA) | 21 | Sucrase-isomaltase (SI) | 9 |
| Nitric oxide synthase 3(NOS3) | 44 | BCL2 apoptosis regulator (BCL2) | 21 | Alkaline phosphatase, intestinal (ALPI) | 8 |
| Ras’s homolog family member A(RHOA) | 43 | Baculoviral IAP repeat containing 5(BIRC5) | 21 | Microsomal glutathione S-transferase 1(MGST1) | 8 |
| Sp1 transcription factor (SP1) | 42 | Checkpoint kinase 2(CHEK2) | 21 | Rhodopsin (RHO) | 8 |
| Hypoxia inducible factor 1 subunit alpha (HIF1A) | 41 | Cathepsin B (CTSB) | 21 | APC regulator of WNT signaling pathway(APC) | 7 |
| Intercellular adhesion molecule 1(ICAM1) | 41 | Cytochrome P450 family 1 subfamily A member 1(CYP1A1) | 21 | Coagulation factor VII(F7) | 7 |
| Nuclear receptor subfamily 3 group C member 1(NR3C1) | 41 | Glial fibrillary acidic protein (GFAP) | 21 | Heat shock transcription factor 1(HSF1) | 7 |
| Amyloid beta precursor protein (APP) | 40 | Maltase-glucoamylase (MGAM) | 21 | 5-hydroxytryptamine receptor 3A(HTR3A) | 7 |
| Brain derived neurotrophic factor (BDNF) | 39 | Prolyl 4-hydroxylase subunit beta(P4HB) | 21 | Cytochrome p450 oxidoreductase (POR) | 7 |
| MCL1 apoptosis regulator (MCL1) | 38 | Raf-1 proto-oncogene (RAF1) | 21 | Succinate dehydrogenase complex flavoprotein subunit A(SDHA) | 7 |
| caspase 9(CASP9) | 37 | Cyclin D2(CCND2) | 20 | Solute carrier family 6 member 1(SLC6A1) | 7 |
| C-reactive protein (CRP) | 37 | Fatty acid binding protein 1(FABP1) | 20 | Aldo-keto reductase family 1 member C1(AKR1C1) | 6 |
| Poly (ADP-ribose) polymerase 1(PARP1) | 37 | Interferon regulatory factor 1(IRF1) | 20 | Cholinergic receptor muscarinic 2(CHRM2) | 6 |
| Sterol regulatory element binding transcription factor 1(SREBF1 | ) 37 | Integrin subunit beta 2(ITGB2) | 20 | Growth hormone receptor (GHR) | 6 |
| Caveolin 1(CAV1) | 36 | Paraoxonase 1(PON1) | 20 | Transient receptor potential cation channel subfamily M member 2(TRPM2) | 6 |
| Glycogen synthase kinase 3 beta (GSK3B） | 36 | Erb-b2 receptor tyrosine kinase 3(ERBB3) | 19 | Alcohol dehydrogenase 1B (class I), beta polypeptide(ADH1B) | 5 |
| Progesterone receptor (PGR) | 36 | Hexokinase 2(HK2) | 19 | D-amino acid oxidase(DAO) | 5 |
| Superoxide dismutase 2(SOD2) | 36 | Haptoglobin (HP) | 19 | Gamma-aminobutyric acid type A receptor subunit alpha1(GABRA1) | 5 |
| Lipoprotein lipase (LPL) | 35 | Acetylcholinesterase (ACHE) | 18 | Thromboxane A2 receptor (TBXA2R) | 5 |
| Cyclin B1(CCNB1) | 34 | Cell division cycle 25C(CDC25C) | 18 | Carnitine O-acetyltransferase (CRAT) | 4 |
| PPARG coactivator 1 alpha (PPARGC1A) | 32 | Inhibitor of nuclear factor kappa B kinase subunit beta (IKBKB) | 18 | Gephyrin (GPHN) | 4 |
| RB transcriptional corepressor 1(RB1) | 32 | Inhibitor of nuclear factor kappa B kinase regulatory subunit gamma (IKBKG | ) 18 | Hemoglobin subunit beta (HBB) | 4 |
| Vascular cell adhesion molecule 19VCAM1） | 32 | Microsomal triglyceride transfer protein (MTTP) | 18 | Solute carrier family 22 member 5(SLC22A5) | 4 |
| Aryl hydrocarbon receptor (AHR) | 31 | recombinase (RAD51) | 18 | Fc fragment of IgE receptor II(FCER2) | 3 |
| Low density lipoprotein receptor (LDLR) | 31 | Aldo-keto reductase family 1 member (BAKR1B1) | 17 | Glycine amidinotransferase (GATM) | 3 |
| NFKB inhibitor alpha (NFKBIA) | 31 | Caspase 2(CASP2) | 17 | Aldehyde dehydrogenase 5 family member A1(ALDH5A1) | 2 |
| Solute carrier family 2 member 4(SLC2A4) | 30 | Kallikrein related peptidase 3(KLK3) | 17 | Malic enzyme 2(ME2) | 2 |
| Mitogen-activated protein kinase 10(MAPK10) | 29 | Ras association domain family member 1(RASSF1) | 17 | Peptidylglycine alpha-amidating monooxygenase(PAM) | 2 |
| Nuclear factor, erythroid 2 like 2(NFE2L2) | 29 | Spi-1 proto-oncogene (SPI1) | 17 | Solute carrier family 7 member 7(SLC7A7) | 2 |
| Caspase 1(CASP1) | 28 | Glutamate ionotropic receptor AMPA type subunit 2(GRIA2) | 16 | Abl interactor 1(ABI1) | 1 |
| Estrogen receptor 2(ESR2) | 28 | Oxidized low density lipoprotein receptor 1(OLR1) | 16 | BRCA1 associated protein 1(BAP1) | 1 |
| Fatty acid synthase (FASN) | 28 | Selectin E (SELE) | 16 | Iodothyronine deiodinase 1(DIO1) | 1 |
| Hexokinase 1(HK1) | 27 | Sterol regulatory element binding transcription factor 2(SREBF2) | 16 | Solute carrier family 25 member 10(SLC25A10) | 1 |
| heat shock protein family B (small) member (HSPB1) | 27 | Dopamine receptor D2(DRD2) | 15 | Telomerase associated protein 1(TEP1) | 1 |
| Insulin like growth factor 2(IGF2) | 27 | Transient receptor potential cation channel subfamily V member 1(TRPV1) | 15 | Tubulin beta 1 class VI(TUBB1) | 1 |
